# Risk Factors of Overweight and Obesity among High School Students in Bahir Dar City, North West Ethiopia: School Based Cross-Sectional Study

**DOI:** 10.1155/2015/294902

**Published:** 2015-12-01

**Authors:** Zelalem Alamrew Anteneh, Molla Gedefaw, Kidist Nigatu Tekletsadek, Meseret Tsegaye, Dagmawi Alemu

**Affiliations:** ^1^School of Public Health, College of Medicine and Health Science, Bahir Dar University, P.O. Box 79, Bahir Dar, Ethiopia; ^2^Disease Prevention and Control Directorate, Federal Ministry of Health, P.O. Box 1234, Addis Ababa, Ethiopia; ^3^Department of Environmental Health and Safety, Ethiopian Electric Power Corporation, P.O. Box 1233, Bahir Dar, Ethiopia; ^4^Department of Mothers and Child Care, Bahir Dar Textile Factory Clinic, P.O. Box 23, Bahir Dar, Ethiopia; ^5^Felege Hiwot Referral Hospital, P.O. Box 209, Bahir Dar, Ethiopia

## Abstract

*Background*. Overweight and obesity are risk factors for diet-related noncommunicable diseases. These diseases are the fifth leading risks for global deaths. Virtually, all age groups are affected from consequences of overweight and obesity.* Methods*. Cross-sectional study was conducted among 431 school adolescents. Data were collected using self-administered questionnaire and physical measurements. The sex and age specific BMI was computed using WHO Anthroplus software and the data were analyzed using bivariate and multivariable logistic regression analysis.* Results*. The magnitudes of overweight and obesity were 12.3% and 4.4%, respectively, and the combined prevalence of overweight and obesity together was 16.7%. Three-fourths of the respondents (74.7%) had healthy body mass index; however, 8.6% were underweight. Sex, frequency of eating food out of home, school type, family monthly income, family having vehicle, vigorous physical activity, and frequency of vigorous physical activity were statistically significant predictors of overweight and obesity.* Conclusion.* The problems of overweight and obesity are taking place while students are still under the risk of underweight. Several factors were correlated with overweight and obesity. Therefore, interventions targeting gender, frequency of eating food out of home, vigorous activities, and frequency of doing vigorous physical activity are recommended.

## 1. Background

Overweight and obesity are excessive fat accumulations in the body which are linked to serious diet-related noncommunicable diseases that affect human health [1]. Globally, obesity is more than twice folded since 1980; more than 1.5 billion adults were overweight and obese. Overweight and obesity are the fifth leading risks for global deaths and are major contributor to the leading killer diseases worldwide, including diabetes, heart disease, and some cancers [2].

Substantial numbers of literatures have been emerged to show that overweight and obesity are major public health challenges to the developing nations causing morbidities and mortalities. Besides, overweight and obesity are causing the health care costs to be substantial [2–4].

Virtually, all age groups and socioeconomic classes of the population are affected by the consequences of overweight and obesity [5]. Adolescents are also one of vulnerable groups to overweight and obesity that could result in premature deaths and disabilities in adulthood. In addition to increased future risks, obese children experience breathing difficulties, increased risk of fractures, hypertension, and early markers of cardiovascular disease [1, 6, 7].

Worldwide, an increased intake of energy dense foods and decreased physical activity because of sedentary nature of many forms of work, changing modes of transportation, and expanding urbanization appeared to contribute to the global overweight and obesity [8].

Low and middle income countries are now facing a double burden of nutritional problems, while they are continuing to deal with undernutrition and are also experiencing a rapid upsurge of noncommunicable disease risk factors such as obesity and overweight [2, 9].

It is now common to find undernutrition and obesity existing side-by-side within the same country, the same community, and the same household. Children in low and middle income countries are vulnerable to inadequate nutrition; at the same time, they are exposed to high fat, sugar, salt, energy dense, micronutrient-poor foods, which tend to be lower in cost, but also lower in nutrient quality. These dietary patterns in conjunction with lower levels of physical activity result in sharp increase in childhood obesity while undernutrition issues remain unsolved [10]. Therefore, the aim of this study was to assess the magnitude and associated factors of overweight and obesity among high school adolescents.

## 2. Materials and Methods

### 2.1. Study Design and Period

A cross-sectional survey was conducted among adolescents and youths in June 2014.

### 2.2. Study Setting

This study was conducted among high school students in Bahir Dar city. The city is located in north western Ethiopia. Bahir Dar city is the capital of the Amhara Administrative Region. It is situated at a distance of 565 kilometers from Addis Ababa, the capital city of Ethiopia.

### 2.3. Eligibility Criteria

Students whose age is between 10 and 24 years in the selected schools were eligible for the study.

### 2.4. Sample Size Determination

The sample size of the study was determined using single population proportion formula, by considering 9.4% expected prevalence of overweight and obesity among school adolescents and youths [11]. Assuming any particular outcome to be within 4% marginal error, 95% confidence interval of certainty, design effect of 2, and additional 10% for nonresponse rate, the sample size was determined to be 451 students.

### 2.5. Sampling Procedure

Bahir Dar city has nine high schools; two of the nine were selected randomly. The study participants were selected from target population through multistage sampling techniques. The calculated samples of students were recruited from selected schools based on the proportional number of their students.

#### 2.5.1. Measurements

The outcome of this study was overweight and obesity, based on age and sex specific body mass index (BMI) for students whose age is less than or equal to 19 years and based on the adult BMI calculation for the students whose age is greater than 19 years.

#### 2.5.2. Data Collection Tools and Procedures

The data were collected using self-administrated structured questionnaires and physical measurements such as heights and weights. The questionnaire was adapted from “WHO STEPwise approach to chronic disease risk factor surveillance” and other reviewed literatures [11–13].

Digital weight measuring instrument was used to take weights of adolescents and youths included in the study. Weight measuring scales were checked and adjusted at zero level; the measurements were taken once from each respondent and the records were made to the nearest 0.5 kg.

Height was measured using height measuring board mounted to weight measuring instrument in standing position following the standard steps; the measurements were taken twice from each respondent and the records were made to the nearest 0.5 cm.

Five nurses as data collector and the principal investigators as supervisor were involved in the field work. During the time of data collection, data collectors were assigned in the selected classes. Just before the class starts, the students selected in the given section were given a questionnaire with an envelope and requested to read the consent form carefully to get informed consent.

The data collectors oriented the students to how to fill the questionnaires properly, and at the end every student's weight and height were taken by data collectors. 

### 2.6. Operational Definition

#### 2.6.1. Overweight

BMI for age greater than or equal to 85th percentile but less than 95th percentile according to the WHO Anthroplus software cutoff point was declared overweight.

#### 2.6.2. Obesity

BMI for age greater than or equal to 95th percentile according to the WHO Anthroplus software cutoff point was declared obesity.

### 2.7. Data Quality Management

The questionnaire was initially prepared in English and was translated into Amharic to obtain the required information from the respondents.

The questionnaire was pretest among randomly selected adolescents in the schools that were not included in the main survey.

Besides, training was given to the data collectors on the methods and procedures of taking measurements on weight and height.

## 3. Data Processing and Analysis

Questionnaires were checked for errors and coded and data were entered into SPSS version 16 software package. Age and sex specific body mass index (BMI) was computed using* WHO Anthroplus* software for the students whose age is less than or equal to 19 years, and BMI for students whose age is greater than 19 years was done using the usual BMI calculation (weight in kilograms per height square in meters (kg/m^2^)).

BMI for age greater than or equal to 85th percentile but less than 95th percentile was overweight and BMI for age greater than or equal to 95th percentile was declared obesity according to the WHO Anthroplus software cutoff points for the students of less than or equal to 19 years.

Univariate and bivariate analyses were computed to see the frequency distribution and to test whether there is association/difference between overweight and obesity and selected independent variables, respectively. Factors associated with overweight and obesity on bivariate were identified, and the variables with *P* value of 20% and less were taken to multivariable logistic regression analysis and the model was built with backward elimination.

Finally, 95% confidence interval not containing one with its corresponding *P* values less than 0.05 was considered statistically significant.

## 4. Ethical Consideration

Ethical clearance was obtained from ethical review committee of GAMBY Medical College and was communicated to the high school administration offices in the selected schools in the city. Permissions were secured from school administration offices and informed consent was obtained from students whose age is above 18 years, and for those whose age is less than 18 years consent was obtained from school administration offices.

## 5. Results

### 5.1. Sociodemographic Characteristics of the Study Participants

A total of 451 students completed the questionnaire; of these 20 responses were excluded because of gross incompleteness; the remaining 431 were included in the analysis. Out of the total respondents, 254 (58.9%) were females. The mean age of the study population was 16.88 ± 1.54. About 232 (53.8%) of respondents were from governmental schools and the remaining 199 (46.2%) were from private owned schools. The majority of the study participants (90%) were Amhara by ethnicity.

Regarding the religious affiliation of study participants, more than 80% were Orthodox Christian and the remaining 20% were from other religious groups. Besides, the families of 75 (29.2%) of respondents were reported to own vehicle for transportation (see Table 1).

### 5.2. Eating Habits of High School Students in Bahir Dar City, North West Ethiopia, 2014

According to the findings of this study, 118 (27.4%) of the respondents ate fruit more than four days per week, 60% of the respondents ate fruits from one to three times per week, and the remaining 13.5% of respondents did not eat fruits at all.

Out of the respondents who were reported to eat fruits, 166 (44.3%) reported to eat fruits one time per day, 144 (38.4%) reported to eat fruits two times per day, and the remaining 119 (27.8%) reported to eat fruits more than three times per day.

About 269 (62.2%), 119 (27.8%), and 10% of respondents were reported to eat vegetables one to three, more than three, and zero times per a week, respectively. Out of the respondents who reported to consume vegetables, 158 (46.7%), 177 (45.6%), and 53 (13.7%) consumed vegetables one time, two times, and three and more times, respectively, in their diet on the day they take vegetables.

The study also indicated that 189 (43.9%), 189 (43.9, and 28 (6.5%) of the respondents were reported to use vegetable, sesame seed, and mixed type of oil in their usual diet.

Regarding frequency of eating out of home, the study indicated that 194 (45%), 53 (12.5%), and 42 (9.7%) of the respondents were reported to eat one to two times, three to five times, and more than five times per week, like in restaurants and hotels.

Besides, 356 (82.6%) of the respondents were eating snacks between their main meals; 338 (87.3%) and 19 (12.3%) of them revealed to eat one to two and three to four times on average per day, respectively. The findings of this study also indicated that 369 (85.6%) and 147 (33.2%) of the respondents were reported to eat while they were watching TV and studying, respectively (Table 2).

### 5.3. Physical Activities of High School Students in Bahir Dar City, 2014

Regarding physical activities of students in Bahir Dar city, the current study revealed that only 149 (34.6%) of them were doing vigorous physical activity. Out of students who were doing vigorous activities, 57 (64.8%), 28 (31.8%), and 3 (3.4%) were doing vigorous activities one to three, four to six, and seven days per week, respectively.

Three hundred thirty-eight (90%) of students were doing moderate physical activities per week; 138 (35.6%), 145 (37.3%), and 105 (27.1%) of the students were doing moderate physical activities one to three, four to six, and seven days per week, respectively.

About 163 (37.8%), 40 (9.3%), 183 (42.5%), and 45 (10.4%) of the students were using taxi, bicycle, feet, and occasionally vehicle, respectively, while they were going to school and back home (Table 3).

### 5.4. Physical Measurements of High School Students in Bahir Dar City, North West Ethiopia, 2014

The findings of this study indicated that the mean height of the respondents was 1.593 with standard deviation of 0.0996 meters. About one-fourth (25.8%) of the respondents have height less than 1.51 meters. The mean weight of the respondents was 53.25 kg with standard deviations of 9.02 kg.

**Figure 1:**
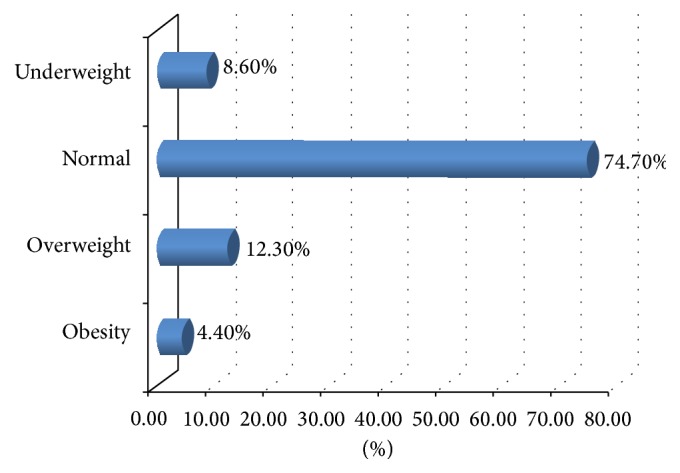
BMI of high school students in Bahir Dar city, North West Ethiopia, 2014.

The prevalence of overweight and obesity was 12.3% and 4.4%, respectively, resulting in the prevalence of overweight and obesity together, 16.7% among school adolescents (see Table 4). Out of the total students involved in the study, proportion of students which showed normal body mass index were 74.7%, whereas 8.6% of high school students were underweight (Figure 1).

### 5.5. Factors Associated with Overweight and Obesity among High School Students in Bahir Dar City, North West Ethiopia, 2014

Bivariate and multivariable logistic regression analyses were computed; in the bivariate analysis sex, school grade level, type of school, family monthly income, presence of family vehicle, frequency of eating snack per day, eating while watching TV/film, frequency of taking soft drinks per week, doing vigorous physical activity, and frequency of doing vigorous physical activity per week were found to be significant at *P* value of 0.2 levels (see Table 5).

In the multivariable logistic regression analysis sex, school type, family monthly income, presence of family vehicle, frequency of eating food out of home, doing vigorous activity, and frequency of doing vigorous physical activity were statically significant predictors of overweight and obesity among high school students at *P* value of 0.05 levels.

The sex of students was significant predictor of overweight and obesity among school students (AOR = 1.93, 95% CI: 1.06–3.51). Students who were attending their education in private schools were found to be more likely to report overweight and obesity as compared to students attending governmental schools (AOR = 1.80, 95% CI: 1.03–3.15).

Respondents from a family whose income is of 251 to 400 and greater than 400 USD were about 2.66 and 4 times more likely to develop overweight and obesity compared to students from family whose monthly income is less than 100 USD, respectively (AOR = 2.66, 95% CI: 1.18–6.02, and 4.00, 95% CI: 1.84–8.72).

The odds of overweight and obesity among students from family having vehicle were more than twice compared to those from family not having vehicle (AOR = 2.07, 95% CI: 1.08–3.94).

Respondents who eat three to five and more than five times per week out of home were more than three and nineteen times likely to develop overweight and obesity than those who eat out of home less than three times a week (AOR = 3.04, 95% CI: 1.97–9.49 and 19.83, 95% CI: 3.96–99.23), respectively.

Besides, respondents who did not do vigorous physical activity were more than four times likely to develop overweight and obesity compared to those who did vigorous physical activity (AOR = 4.09, 95% CI: 1.26–13.32). Moreover, respondents who did vigorous physical activity less than three times a week were more than four times likely to develop overweight and obesity compared to those who did vigorous physical activity more than six times a week (AOR = 4.30, 95% CI: 1.32–14.05) (Table 6).

## 6. Discussion

According to the findings of this study the prevalence of overweight and obesity was 12.3% and 4.4%, respectively, resulting in the prevalence of overweight and obesity together, 16.7%. The magnitude of overweight in the current study was in accordance with a study done among students in Port Said city in Egypt where the prevalence of overweight was 17.7%, whereas the prevalence of obesity in our study was much lower than the study done in Port Said city, where the prevalence of obesity was 13.5% [14].

The findings of the current study on magnitude of overweight and obesity were in agreement with a study done in Nigeria among students, where the prevalence rates of overweight and obesity were 11.4% and 2.8%, respectively [15], whereas our finding is not in accordance with other studies in Pakistan, where the prevalence of overweight was 23% [16]. Besides, the findings of this study were lower than that of a study done in united states among children and adolescents, where the prevalence of overweight and obesity was 21–24% and 16–18%, respectively [17]. The reason for the variability may be attributed to different factors including the sociodemographic and economic variability.

Regarding the analytic component of this study, the odds of developing overweight and obesity among females were higher than males. This was in accordance with a study done in African countries that female students were more than twice to be at risk of overweight and obesity compared to male students [3, 17, 18].

The findings of the current study indicated that as the frequency of eating out of home increases the risk of overweight and obesity increases. The reason might be an increase in the episodes of eating associated high nutritional intake; in turn this can increase the occurrence of overweight and obesity [19, 20].

The findings of this study revealed that students who were attending private owned schools had higher risks of overweight and obesity compared to those in governmental schools. This was in line with other similar studies in different region of the globe, where adolescents learning in private schools reported higher risks of overweight and obesity [12, 21]. This might be because adolescents in private schools usually come from families with higher socioeconomic groups which in turn might be exposed to high energy dense foods. In addition, a student from high economic classes usually uses vehicles for transportation. Moreover, adolescents in private school may also have less restriction on food and snack choices compared with those in public schools [22].

The findings of this study also indicated that family monthly income was significantly associated with the overweight and obesity. Accordingly, students from family with monthly income of $251 to 400 and greater than $400 were more than two and four times likely to develop overweight and obesity compared to those from family of less than $100 monthly family income. This is supported with other studies, where family income is associated with overweight and obesity [12, 23, 24].

This might be because students from family of high income group may take high energy dense foods and might be doing less exercise and even use vehicles for transportation; this study also showed that adolescents from family having vehicle for transportation were in the high income group.

Students from family having vehicle were more likely to report overweight and obesity compared to their counterparts, indicating that adolescents from family owning vehicle had more than double risk of overweight or obesity. This finding is in accordance with a study where family vehicle is associated with the occurrence of overweight and obesity [25].

Moreover, respondents who did not have vigorous physical activities were more likely to develop overweight and obesity compared to respondents who were doing vigorous physical activities. This might be because doing vigorous physical activities burns off body fat and is associated with less risk of overweight or obesity. This is in line with other studies, where vigorous physical activities were associated with occurrence of overweight and obesity [26, 27].

Moreover, frequency of doing vigorous physical activity was statistically associated with the occurrence of overweight and obesity. Respondents who were doing vigorous physical activity less frequently were more likely to report overweight and obesity compared to those who were doing it frequently; this is also supported by similar studies [28, 29].

## 7. Limitations of the Study

Even though this study addressed very important variables that affect overweight and obesity, skin fold measurements were not done, and variables on behavioral factors like smoking and alcohol consumption, biochemical factors, parental weight status, and nutritional knowledge of the respondents were not covered.

## 8. Conclusion

The prevalence of overweight and obesity among school adolescents was 12.3% and 4.4%, respectively. The overall prevalence of overweight and obesity together was 16.7%.

Factors such as sex, school type, family monthly income, having a family vehicle, frequency of eating out of home per week, and doing vigorous activity and the frequency of doing vigorous physical activity per week were independently correlated with overweight and obesity.

Therefore, the regional government, the schools, and the families need to collaborate to improve the health of the students.

Nutritional based interventions need to be promoted and implemented by concerned bodies to prevent future risks linked to overweight and obesity.

Besides, frequency of eating food out of home needs attention to prevent overweight and obesity. Moreover, physical activities should be promoted to engage students in physical exercises in order to prevent fat accumulation.

## Figures and Tables

**Table 1:** Sociodemographic characteristics of the high school students in Bahir Dar city, North West Ethiopia, 2014.

Variables	Categories	Frequencies (*N* = 431)	Percentages
Sex	Male	177	41.1
	Female	254	58.9

Age in years	<16	187	43.4
	16-17	100	22.5
	17.1–18	91	21.8
	>18	53	12.3

School type	Governmental	232	53.8
	Private	199	46.2

Father's occupation	Civil servant	170	39.4
	NGO	69	16
	Merchant	119	27.6
	Farmer	59	13.7
	Retired	14	3.2

Mother's occupation	Civil servant	131	30.4
	NGO	22	5.1
	Merchant	90	20.9
	Farmer	23	5.3
	Retired	6	1.4
	House wife	159	36.9

Mother's education	Illiterate	28	6.5
	Read and write	36	8.4
	Grade 1 to 8	64	14.8
	Grade 9 to 12	104	42.1
	Diploma and above	199	46.2

Family own vehicle	Yes	75	17.4
	No	356	82.6

Family monthly income in USD	<100	126	29.2
	100–250	128	29.7
	251–400	72	16.7
	>400	81	18.8

**Table 2:** Eating habits of high school students in Bahir Dar city, North West Ethiopia, 2014.

Variables	Categories	Frequencies	Percentages
Number of days of fruit intake/week (*n* = 431)	Zero	58	13.5
	One to three times	255	59.1
	More than three times	118	27.4

Number of times of fruit intake on the day fruit were taken (*n* = 375)	One	166	44.3
	Two	144	38.4
	Three and more times	65	17.3

Number of days of vegetables intake/week (*n* = 431)	Zero	43	10.0
	One to three times	269	62.2
	More than three times	119	27.8

Number of times of vegetables intake on the day fruit were taken (*n* = 388)	One	158	46.7
	Two	177	45.6
	Three and more times	53	13.7

Average times of eating food out of home per week (*n* = 431)	One to two times	194	45
	Three to five times	53	12.5
	More than five times	42	9.7

Eaten snack (*n* = 431)	Yes	356	82.6
	No	75	17.4

Number of times snack was eaten per day (*n* = 356)	One to two times	338	87.3
	Three to four times	19	12.6

Number of times of normal food eaten other than snack/day (*n* = 431)	Two	86	20
	Three	298	69.1
	More than three times	47	10.9

The food eaten other than main food (*n* = 615)	Cake	147	23.9
	Biscuits	300	48.8
	Ice cream	73	11.8
	Chocolate	95	15.4

Eating when studying (*n* = 431)	Yes	147	33.2
	No	288	66.8

Number of times soft drink was taken per week (*n* = 276)	1 to 2 times	201	72.8
	3 to 5 times	66	23.9
	Greater than 5 times	9	3.3

**Table 3:** Physical activities of high school students in Bahir Dar city, North West Ethiopia, 2014.

Variables	Categories	Frequencies	Percentages
Vigorous activity (*n* = 431)	Yes	149	34.6
	No	282	65.4

Number of days vigorous activity was done/week (*n* = 88)	1 to 3	57	64.8
	4 to 6	28	31.8
	7 days	3	3.4

Moderate physical activity (*n* = 431)	Yes	388	90
	No	43	10

Number of days moderate activities were done per week (*n* = 388)	1 to 3	138	35.6
	4 to 6	145	37.3
	7 days	105	27.1

Means of transport to and from school	Car/taxi/	163	37.8
	Bicycle	40	9.3
	Feet	183	42.5
	Sometimes vehicle	45	10.4

Vigorous sport	Yes	188	43.6
	No	243	56.4

Number of days vigorous sport was done per week (*n* = 188)	1 to 3	143	76.1
	4 to 6	31	16.4
	7 days	14	7.5

Number of hours spent in TV watching (*n* = 431)	1 to 2	142	34.5
	2.1 to 3	80	19.5
	3.1 to 4	88	21.4
	>4	101	24.6

**Table 4:** Physical measurements of high school students in Bahir Dar city, North West Ethiopia, 2014.

Variables	Classification	Frequency (*N* = 431)	Percentages
Height (mean = 1.59 m)	<1.51 m	111	25.8
	1.51–1.60 m	139	32.3
	1.61–1.65 m	81	18.8
	>1.65 m	100	23.2

Weight (mean = 53.25 kg)	<47 kg	113	26.2
	47–52 kg	115	26.7
	52.1–58 kg	97	22.5
	>52 kg	106	24.6

BMI (mean = 21.02 kg/m^2^)	<18 kg/m^2^	37	8.6
	18–24 kg/m^2^	322	74.7
	25–30 kg/m^2^	53	12.3
	>30 kg/m^2^	19	4.4

Overweight (BMI 25–30 kg/m^2^)	Yes	53	12.3
	No	378	87.7

Obesity (>30 kg/m^2^)	Yes	19	4.4
	No	412	95.6

Overweight and obesity	Yes	72	16.7
	No	359	83.3

Note: the height and weight were categorized using quartile system.

**Table 5:** The correlation between selected predictor variables and overweight and obesity on crude effect among high school students in Bahir Dar city, 2014.

Variables	Categories	Overweight and obesity (*n* = 431)		COR (95% CI for OR)	*P* value
		Yes	No		
Sex	Male	22	155	1.00	
	Female	50	204	1.73 (1.00–2.97)	0.049

School grade level	Grade 9	27	193	1.00	0.013
	Grade 11	45	166	1.94 (1.15–3.26)	

School type	Governmental	29	203	1.00	0.012
	Private	49	156	1.93 (1.15–3.23)	

Family monthly income	<100 USD	13	113	1.00	
	100–250 USD	11	117	0.82 (0.35–1.90)	<0.001
	251–400 USD	16	56	2.48 (1.12–5.52)	
	>400 USD	27	54	4.35 (2.08–9.08)	

Having family vehicle	Yes	22	53	2.54 (1.42–4.54)	0.002
	No	50	306	1.00	

Frequency of eating food out of home	One to two	28	166	1.00	
	Three to five times	14	39	2.13 (1.03–4.42)	<0.001
	Greater than five times	20	22	5.39 (2.61–11.14)	

Frequency of eating snack/day	One to two	37	250	1.00	<0.001
	Three to four	32	43	5.03 (2.84–8.92)	

Eat when watching TV/film	Yes	70	299	7.02 (1.68–29.43)	0.008
	No	2	60	1.00	

Frequency of taking soft drink/week	One to two	29	172	1.00	
	Three to five	13	53	1.446 (0.71–3.00)	0.014
	Greater than five	5	4	7.41 (1.88–29.25)	

Vigorous activity	Yes	15	134	1.00	0.008
	No	57	225	2.26 (1.23–4.16)	

Frequency of vigorous physical activity/week	One to three	23	50	2.80 (1.44–5.47)	0.008
	Four to six	11	54	1.24 (0.56–2.73)	
	Greater than six	22	134	1.00	

**Table 6:** The association between selected predictor variables and overweight and obesity among high school students in Bahir Dar city, North West Ethiopia, 2014.

Variables	Categories	Overweight and obesity (*n* = 431)		AOR (95%CI for OR)	*P* value
		Yes	No		
Sex	Male	22	155	1.00	0.032^*∗*^
	Female	50	204	1.93 (1.06–3.51)	

School grade level	Grade 9	27	193	1.00	0.265
	Grade 11	45	166	1.42 (0.77–2.63)	

School type	Governmental	29	203	1.00	0.039^*∗*^
	Private	49	156	1.80 (1.03–3.15)	

Family monthly income	<100 USD	13	113	1.00	
	100–250 USD	11	117	0.87 (0.37–2.06)	<0.001^*∗*^
	251–400 USD	16	56	2.66 (1.18–6.02)	
	>400 USD	27	54	4.00 (1.84–8.72)	

Having family vehicle	Yes	22	53	2.07 (1.08–3.94)	0.028^*∗*^
	No	50	306	1.00	

Frequency of eating food out of home	One to two	28	166	1.00	0.001^*∗*^
	Three to five times	14	39	3.04 (1.97–9.49)	
	Greater than five times	20	22	19.83 (3.96–99.23)	

Frequency of eating snack/day	One to two	37	250	1.00	0.078
	Three to four	32	43	2.53 (0.90–7.09)	

Eating when watching TV/film	Yes	70	299	2.15 (0.20–22.86)	0.64
	No	2	60	1.00	

Frequency of taking soft drink/week	One to two	29	172	1.00	
	Three to five	13	53	0.34 (0.04–3.19)	0.25
	Greater than five	5	4	0.36 (0.03–3.88)	

Vigorous activity	Yes	15	134	1.00	0.019^*∗*^
	No	57	225	4.09 (1.26–13.32)	

Frequency of vigorous physical activity/week	One to three	23	50	4.30 (1.32–14.05)	0.04^*∗*^
	Four to six	11	54	1.08 (0.28–4.02)	
	Greater than six	22	134	1.00	

Note: the asterisk (*∗*) shows that the variable is statistically associated with the dependent variable in the multivariable logistic regression analysis.
